# Coping Experiences of Middle-Aged Female Survivors of Sex-Specific Cancer

**DOI:** 10.1093/geroni/igaf122.3201

**Published:** 2025-12-31

**Authors:** Sunui Shin, Hyun-E Yeom, Misook Jung, Eunyoung Park

**Affiliations:** Chungnam National University, Daejeon, Republic of Korea; Chungnam National University, Daejeon, Republic of Korea; Chungnam National University, Daejeon, Republic of Korea; Chungnam National University, Daejeon, Republic of Korea

**Keywords:** Sex-Specific Cancer Survivors, Identity Change, Qualitative research

## Abstract

Women diagnosed with sex-specific cancers encounter multifaceted physical and psychological challenges throughout survivorship, particularly concerning their perception of femininity. Alongside aging, middle-aged female survivors must navigate role transitions as mothers and shifts in spousal relationships, leading to broader family and social changes. This study explored their coping experiences, focusing on identity and role transitions. A phenomenological qualitative approach was employed, using purposive sampling to recruit 10 female cancer survivors. The participants had a mean age of 49.1 years, including four diagnosed with breast cancer, five with ovarian cancer, and one with uterine cancer. Data were collected through semi-structured, in-depth interviews and analyzed using Colaizzi’s phenomenological method. Four key themes emerged: (1) Threatened Femininity – participants experienced a disrupted sense of femininity due to hair loss, breast changes, and ovarian removal; (2) Concerns and Guilt for Their Children – anxiety about leaving their children behind and guilt over the hereditary nature of cancer; (3) Changes in Marital Relationships – physical changes challenged marital life, yet spousal care and emotional support strengthened relationships; and (4) Shifts in Life Perspectives – despite emotional distress, participants developed a greater appreciation for daily life, leading to renewed purpose. These findings highlight the need to understand the transitions experienced by middle-aged female cancer survivors. They emphasize the importance of a comprehensive approach that considers physical, identity, relational, and life changes to support survivors better.

